# Sensory Response System of Social Behavior Tied to Female Reproductive Traits

**DOI:** 10.1371/journal.pone.0003397

**Published:** 2008-10-14

**Authors:** Jennifer M. Tsuruda, Gro V. Amdam, Robert E. Page

**Affiliations:** 1 Department of Entomology, University of California Davis, Davis, California, United States of America; 2 School of Life Sciences, Arizona State University, Tempe, Arizona, United States of America; 3 Department of Chemistry, Biotechnology and Food Science, Norwegian University of Life Sciences, Aas, Norway; University of Lausanne, Switzerland

## Abstract

**Background:**

Honey bees display a complex set of anatomical, physiological, and behavioral traits that correlate with the colony storage of surplus pollen (pollen hoarding). We hypothesize that the association of these traits is a result of pleiotropy in a gene signaling network that was co-opted by natural selection to function in worker division of labor and foraging specialization. By acting on the gene network, selection can change a suite of traits, including stimulus/response relationships that affect individual foraging behavior and alter the colony level trait of pollen hoarding. The ‘pollen-hoarding syndrome’ of honey bees is the best documented syndrome of insect social organization. It can be exemplified as a link between reproductive anatomy (ovary size), physiology (yolk protein level), and foraging behavior in honey bee strains selected for pollen hoarding, a colony level trait. The syndrome gave rise to the forager-Reproductive Ground Plan Hypothesis (RGPH), which proposes that the regulatory control of foraging onset and foraging preference toward nectar or pollen was derived from a reproductive signaling network. This view was recently challenged. To resolve the controversy, we tested the associations between reproductive anatomy, physiology, and stimulus/response relationships of behavior in wild-type honey bees.

**Methodology/Principal Findings:**

Central to the stimulus/response relationships of honey bee foraging behavior and pollen hoarding is the behavioral trait of sensory sensitivity to sucrose (an important sugar in nectar). To test the linkage of reproductive traits and sensory response systems of social behavior, we measured sucrose responsiveness with the proboscis extension response (PER) assay and quantified ovary size and *vitellogenin* (yolk precursor) gene expression in 6–7-day-old bees by counting ovarioles (ovary filaments) and by using semiquantitative real time RT-PCR. We show that bees with larger ovaries (more ovarioles) are characterized by higher levels of *vitellogenin* mRNA expression and are more responsive to sucrose solutions, a trait that is central to division of labor and foraging specialization.

**Conclusions/Significance:**

Our results establish that in wild-type honey bees, ovary size and *vitellogenin* mRNA level covary with the sucrose sensory response system, an important component of foraging behavior. This finding validates links between reproductive physiology and behavioral-trait associations of the pollen-hoarding syndrome of honey bees, and supports the forager-RGPH. Our data address a current evolutionary debate, and represent the first direct demonstration of the links between reproductive anatomy, physiology, and behavioral response systems that are central to the control of complex social behavior in insects.

## Introduction

How do complex phenotypes evolve? To answer this question is a central challenge in evolutionary biology that becomes all the more difficult when complex phenotypes are social [1]. Recent progress has been made in studies of honey bee foraging behavior, a social trait (reviewed by [2]–[4]). Experiments on wild-type [5]–[9] and selected honey bee strains, coupled with genetic mapping [10]–[18], have revealed complex phenotypic architectures that are linked by epistasis and pleiotropy. Central to the phenotypic architecture is a suite of correlated traits at different levels of biological organization associated with a colony-level activity, the collection and storage of surplus pollen [3]–[6]. This suite of traits has been called the pollen-hoarding syndrome because its correlations were initially revealed through studies of bees that were selected for their pollen-hoarding behavior [16].

Bi-directional colony-level selection resulted in two strains, the high pollen-hoarding strain and the low pollen-hoarding strain, that differ dramatically in the amount of surplus pollen they store [16]. In addition to colony differences in stored pollen, the selection caused worker bees (essentially sterile ‘helpers’) from the two strains to diverge for multiple anatomical, physiological, and behavioral traits. For example, compared to workers from the low pollen-hoarding strain, high strain bees initiate foraging earlier in life, are more likely to collect pollen, collect heavier loads of pollen and lighter loads of nectar, tend to collect nectar with lower concentrations of sucrose, are more responsive to gustatory stimuli (sucrose), have larger-sized ovaries (more ovary filaments), and as young adults high strain bees have higher fat body mRNA expression levels and hemolymph (blood) titers of the yolk precursor vitellogenin (reviewed by [2]–[4]).

All worker honey bees are female but have ovaries that are greatly reduced in size compared to the queen [19]. Though normally inhibited from egg-laying, worker bees synthesize vitellogenin throughout the first weeks of adult life (reviewed by [20], [21]). Honey bee vitellogenin interacts in a mutually repressive feedback-loop with juvenile hormone (JH) [17], [22], [23], a developmental and reproductive hormone (reviewed by [24]) that also affects behavior [25], [26]. We hypothesized that co-option of the reproductive regulatory network of honey bee ancestors, including the interface between vitellogenin and JH, occurred with the evolution of an advanced division of labor system (reviewed by [27]). We suggested that selection on this network enabled, or reinforced, a temporal differentiation between worker bees so they first labor inside the nest and later forage [22]. We further suggested that the network gave rise to a behavioral segregation of foragers with biases for collecting nectar or pollen [11] and that this shared regulatory origin may explain the observed linkage between the age at foraging onset, the foraging specialization, and the reproductive anatomy and physiology of worker honey bees [5]. We called the underlying evolutionary framework the Reproductive Ground Plan Hypothesis [11]. The hypothesis was build from our cumulative data on the pollen-hoarding syndrome [2], [3], [27] and was inspired by the Ovarian Ground Plan Hypothesis (OGPH) of West-Eberhard, which suggests that evolution of a division of labor between within-nest and foraging tasks has a reproductive basis in social insects [1], [28].

Oldroyd and Beekman [29] recently proposed that the RGPH be called the forager-RGPH to better distinguish it from the OGPH of West-Eberhard. Intuitively, the forager-RGPH, like the OGPH, is difficult to test directly using extant social bees because the hypothesized reproductive basis of their advance division of labor has been acted upon by colony-level selection. However, support for the hypothesis may be found by studying relationships between worker reproductive anatomy, physiology, and division of labor and foraging specialization [5]. Studies of wild type bees and bees selected for pollen hoarding behavior demonstrate that variation in the reproductive anatomy and physiology of workers covaries with different sensory states and behavioral biases, including the onset of foraging behavior and foraging for pollen and nectar [5], [11], [29]. A study of more than 500 worker bees from four wild-type colonies established that bees with more ovarioles forage earlier in life and bias their foraging effort toward pollen and accept nectar of lower sucrose concentrations, as repeatedly demonstrated for high pollen-hoarding strain bees [5]. Down regulation of *vitellogenin* gene activity by RNA interference (RNAi), furthermore, affects several components of the pollen-hoarding syndrome in wild-type workers, specifically the age of foraging onset, foraging specialization, and sensitivity to sucrose [7], [30]. Similar endocrine integration of sensory responses, feeding behavior, and female reproductive traits are present in many taxa [31]–[34], and may be central in biasing the female foraging choice toward essential nutrients, such as protein, in times of egg production and provisioning of young (reviewed by [34]).

Recently, Oldroyd and Beekman challenged the forager-RGPH [29]. They compared ovary size, ovary activation, and foraging behavior of wild-type bees with a strain of bees selected for abnormal reproductive behavior (anarchistic bees). Anarchistic workers express a rare behavioral phenotype that can lay eggs in the presence of a functional queen [35], [36]. This reproductive behavior and its underlying physiology is fundamentally different from that of wild-type workers [35], [37], [38]. Their average ovary size is smaller than low pollen-hoarding strain bees and wild-type [5], [17], [29], yet anarchistic bees have a higher tendency to lay eggs in the presence of a queen [37], suggesting that they are more resistant to pheromonal inhibition of oviposition [39]. Oldroyd and Beekman [29] proposed that if the forager-RGPH holds, anarchistic bees should display the behavioral phenotype of high pollen-hoarders because the anarchistic strain is ‘more reproductive’ than wild-type. However the anarchistic workers failed to show a foraging bias toward pollen and initiated foraging later in life than wild-type bees. Oldroyd and Beekman concluded that they doubted “… the validity of a general association between reproductive potential and division of labor when foraging, modulated by the production of vitellogenin”, p6 [29].

We have previously shown associations between ovary size and foraging behavior in wild-type bees [5]. We have further demonstrated that RNAi-induced reduction of hemolymph levels of vitellogenin results in changes in foraging behavior and the responsiveness to sucrose of wild-type bees, supporting the forager-RGPH. Sucrose responsiveness has repeatedly been shown in wild-type pre-foraging bees to be correlated with their foraging biases for nectar and pollen when they initiate foraging days-to-weeks later [6], [8], [9]. Sucrose responsiveness also is a central component of the pollen-hoarding syndrome [3]. Here we test for the first time the hypothesis that ovary size (ovariole number) in wild type bees correlates with vitellogenin titers and responsiveness to sucrose, a direct test of the assertions of Oldroyd and Beekman [29], discussed above. We predicted that bees with more ovarioles are characterized by elevated levels of *vitellogenin* mRNA and, as a consequence, increased sucrose responsiveness.

## Results

### Ovary size and sucrose responsiveness

We first looked at the association between ovary size and sucrose responsiveness in 6–7 day-old wild-type bees. The proboscis extension response (PER) assay was used to test for sucrose sensitivity using a series of 7 sugar concentrations. Individuals were assigned a gustatory response score (GRS) based on the number of sugar concentrations that elicited a response (proboscis extension); a high GRS reflects a high responsiveness to sucrose (see Materials and Methods [40]).

**Figure 1:**
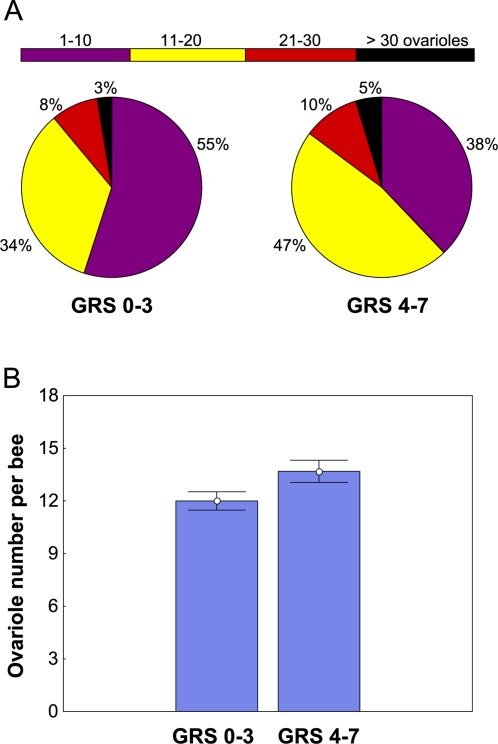
Relationships between Sucrose Responsiveness and Ovary Size in Worker Honey Bees. (A) Pie charts showing the distributions of ovary sizes between bees with low (GRS 0–3, *n* = 141) and high (GRS 4–9, *n* = 149) responsiveness to sucrose. Ovary sizes are given as the total number of ovarioles per bee (i.e., summing over both ovaries). (B) Comparison between the means and standard errors of ovary sizes between bees with low and high sucrose responsiveness. Bees with high GRS scores are characterized by significantly larger ovaries on average.

GRS testing was followed by abdominal dissections to remove the ovaries and count the total number of ovarioles of each individual bee. A dorsal section of the abdominal wall with adhering fat body tissue (the major site of *vitellogenin* gene activity in honey bees [41]) attached was removed and frozen in Trizol reagent for later RNA extraction and *vitellogenin* mRNA quantification (see below). For analysis of the relationship between ovary size and the sucrose response system, we tested for a difference in total ovariole number between the bees with low responsiveness (GRS 0–3) and high responsiveness (GRS 4–7) to sucrose (Fig. 1A). As predicted, we found that workers with high sucrose responsiveness (GRS 4–7, *n* = 141) were characterized by a higher number of ovarioles than the bees with lower sucrose responsiveness (GRS 0–3, *n* = 149, P = 0.037, one-way ANOVA; Fig. 1A–B).

### Ovary size, sucrose responsiveness and *vitellogenin* gene activity

**Figure 2:**
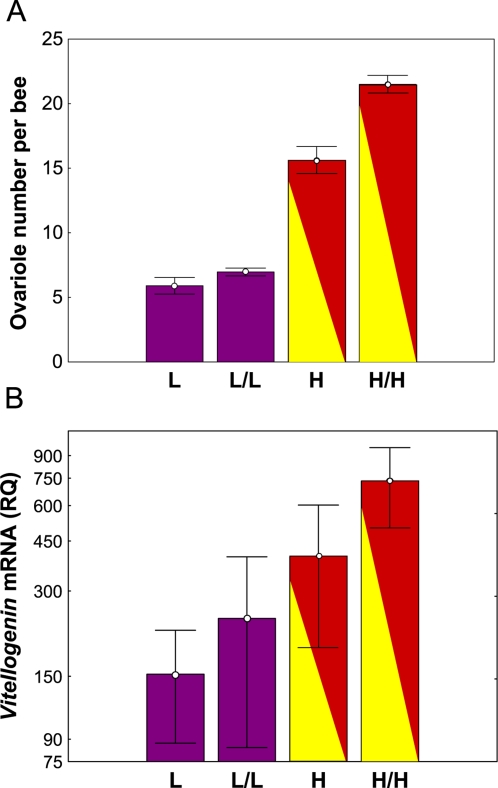
Association of Sucrose Responsiveness, Ovary Size, and *Vitellogenin* mRNA Level in Worker Honey Bees. (A) Means and standard errors of the ovary sizes in the subsets of bees selected from the extreme tails of the ovariole number and GRS and distributions; LL = small ovaries (3–9 ovarioles) and low GRS (0–2), HH = large ovaries (17–29 ovarioles) and high GRS (4–7), *n* = 44 and 43, respectively. L and H were laboratory handling controls that were selected only on the basis of ovary size, GRS was not determined (*n* = 10; ovariole number was 3–9 and 12–23, respectively, H spanned lower ovary sizes than HH as there were not a sufficient number of bees to obtain in the 17–29 range). HH and LL differ significantly for ovariole number (P<0.0001, one-way ANOVA). (B) Means and standard errors of the log-transformed *vitellogenin* mRNA expression level given as a relative quantity (RQ). Bees with large ovaries and high GRS are characterized by significantly higher *vitellogenin* levels on average (P<0.005, one-way ANOVA). The controls show no significant effect of handling.

Next, RNA was extracted from the abdominal tissue samples for the individuals that had scored the extremes of the ovariole number and GRS distributions [11]; bees with low numbers of ovarioles and low sucrose responsiveness (LL) and those with high numbers of ovarioles and high sucrose responsiveness (HH). Using these extreme phenotypes enhanced the power of our analyses and is standard practice in studies where phenotyping is less difficult and less expensive than the genetic or physiological analyses [10], [18]. This resulted in a sample set (Fig. 2A) that had an *a priori* expectation for higher levels of *vitellogenin* gene activity in the samples with high GRS and high ovarioles [23]. The two traits, ovariole number and GRS were not independent. The effect of ovariole number on GRS was expected to be a result of the effect of ovariole number on vitellogenin production. Bees from the HH and LL groups differed significantly for both number of ovarioles (shown in Fig. 2A; X = 21.5 and 7.0, respectively, *n* = 43 and 44, P<0.0001, one-way ANOVA) and GRS (X = 4.9 and 1.5, respectively, P<0.0001).

The amount of *vitellogenin* mRNA was quantified semi-quantitatively using actin as an active reference [11].The analysis documented that bees that on average had high numbers of ovarioles and high sucrose responsiveness (HH) also had higher relative levels of *vitellogenin* mRNA expression than bees with low numbers of ovarioles and low sucrose responsiveness (P = 0.0025; Fig. 2B). Regression analysis showed that GRS explained more of the variance in *vitellogenin* expression (log transformed data) than did ovariole number (GRS: r^2^ = 0.12, *n* = 57, P = 0.004; ovariole number: r^2^ = 0.08, *n* = 57, P = 0.019). Stepwise multiple regression, using both GRS and ovariole number as “independent” variables, demonstrated that only GRS explained a significant amount of the variance in *vitellogenin* mRNA (P = 0.004). This was expected considering the hypothesized causal chain of ovariole number, vitellogenin synthesis, and GRS. In this case, the effects of vitellogenin on GRS shield the effects of ovariole number. The experimental protocol also included a control for laboratory handling. Subsets of bees with high (H) vs. low (L) ovariole number were processed for *vitellogenin* mRNA quantification directly, without being subject to the GRS assay. These controls established that *vitellogenin* mRNA levels are not significantly affected by the procedure used to quantify sucrose responsiveness.

## Discussion

Sucrose responsiveness has been shown repeatedly to correlate with the behavioral components of the pollen-hoarding syndrome (reviewed by [2]). For example, the response to sucrose solution of young wild-type workers can be used to predict their foraging behavior 2–3 weeks later [6], [8], [9]; bees that are more responsive to sucrose are characterized by a bias toward collecting pollen whereas less responsive bees collect more nectar. Furthermore, bees with high sucrose responsiveness initiate foraging earlier in life than those that are less responsive to sucrose and will collect nectar with lower sugar concentration, like high pollen-hoarding strain bees. Average ovary size and vitellogenin levels are also different between strains bi-directionally selected for amount of stored surplus pollen. Compared to the low strain, high pollen-hoarding strain bees have more ovarioles, and as young adults they have higher vitellogenin titers [5], [11], [17]. Ovariole number was shown previously to correlate with the foraging behavior of wild-type workers, those with more ovarioles foraged earlier in life and showed a foraging bias toward pollen and nectar with lower sugar concentrations [5]. *Vitellogenin* gene activity, moreover, has complex and broad effects on honey bee foraging behavior, and modulates sucrose responsiveness [30], foraging onset, and foraging preference toward pollen and nectar [7]. Overall, elevated vitellogenin levels early in life have been linked to a foraging bias toward pollen (reviewed by [27]). Here, we link these associations and demonstrate for the first time in wild-type honey bees that ovary size is associated with *vitellogenin* gene activity and the sucrose response system.

Collectively, these data support the forager-RGPH [5], [11] and stand in opposition to the conclusions of Oldroyd and Beekman [29]. The disparity between their results and the body of work we presented here and previously (see [2], [3] for reviews) may be explained by the lack of sufficient variance in ovariole number in their study. Oldroyd and Beekman reported average ovary sizes of 2.0 and 2.3 ovarioles per ovary for anarchistic bees and wild-type bees, respectively. For comparison, in our previous study [5] colonies had mean worker ovariole counts of 4±2.4 S.D. and 5±3.1 per ovary while in this study they had an average of 6±3.6.

The results presented here support the hypothesis that linkage of reproductive anatomy, physiology, and sensory responses are associated with foraging behavior and may be a general property of behavioral control in worker honey bees, consistent with the forager-RGPH. Also, they demonstrate how complex social behavior (division of labor) may evolve from reproductive regulatory networks that are deeply entrenched in insect development and evolutionary history.

## Materials and Methods

### Bees

Experiments were performed at the University of California, Davis. Initial surveys were conducted to find a wild-type (commercial unselected) source colony with sufficient variation in the number of ovarioles in workers. Once a suitable colony was identified, frames of developing pupae (12 hours prior to emergence) were placed in an incubator overnight. Newly emerged workers were marked on the thorax with a spot of paint (Testors Enamel) to indicate age. Bees emerged for 6 days and each day the set of emerged bees were added to a different unrelated “host” colony. Amdam et al. [11] showed that significant differences in *vitellogenin* mRNA levels could be detected between high and low pollen-hoarding strain bees at ages 5 and 10 days old. In reference to this age-span, we collected the marked wild-type workers as 6–7 day-olds.

### Measurement of Sucrose Responsiveness

Bees were collected from the brood combs and placed individually into cylindrical mesh cages. Each bee was chilled in a refrigerator until the first signs of immobilization, mounted into a small brass tube secured with strips of adhesive tape between the head and thorax and over the abdomen. After recovering, each bee was fed up to 10 µl of 10% sucrose to control for hunger. They were then assayed for the PER by touching the antennae with a droplet of sucrose solution or water using the techniques described by Bitterman et al. [42]. Testing began 30–45 minutes after the last bee of each group was fed. Bees were assayed using water followed by a concentration series of 0.1, 0.3, 1, 3, 10, and 30% sucrose by weight, corresponding to a logarithmic series of −1, −0.5, 0, 0.5, 1, and 1.5. All bees were lined up and each was tested one time, in sequential order, at each of the concentrations, e.g., all were tested at 0.1% first, then all were tested at 0.3%, etc. The inter-stimulus interval varied between 3 to 5 minutes with the number of individuals tested at one time, usually 60 bees per test. A bee was observed to ‘respond’ by fully extending its proboscis when a drop of water or sucrose was touched in turn to each antenna. Assigned gustatory response scores (GRS) ranged from 0 (no response to any concentration) to 7 (response to all concentrations). A subset of the collected bees was not tested for sucrose responsiveness but dissected for ovary size and processed for *vitellogenin* mRNA quantification within two hours of collection.

### Quantification of Ovary Size and *vitellogenin* mRNA

Bees were immobilized in a refrigerator and prepared for dissection. For each bee, the abdomen was separated from the thorax with forceps. Dorsal incisions were made in the abdomen and the right- and left-side ovaries were transferred to a glass slide. The total number of ovarioles was counted under a compound stereomicroscope.

RNA was extracted from the abdominal tissue as described before [11]. For each individual bee, 100 ng of total RNA was analyzed by real-time RT-PCR using the QuantiTect SYBR green RT-PCR kit (Qiagen). For each sample, triplicate reactions were run for *vitellogenin* and *actin*, and one reaction without reverse transcriptase as a non-template cDNA control. Primers were: *vitellogenin* (5 -GTTGGAGAGCAACATGCAGA- 3; 5 -TCGATCCATTCCTTGATGGT- 3) and *actin* (5 -TGCCAACACTGTCCTTTCTG- 3; 5 -AGAATTGACCCACCAATCCA- 3). Relative *vitellogenin* mRNA expression levels were determined by the comparative CT method as described previously [11]. To verify that the SYBR green dye detected only the intended PCR product, all reactions were subject to the heat-dissociation protocol following the final cycle of the PCR.

### Data Analysis

Analyses were performed with JMP (SAS Institute Inc.). Ovariole counts were compared between GRS classes using ANOVA. The *a priori* hypothesis was that GRS class 0–3 has fewer ovarioles. Linear regression was used to estimate r^2^ for the associations of ovariole number and GRS with vitellogenin expression. Gene expression data were log transformed to approximate a normal distribution. ANOVA was again used to test the *a priori* hypothesis that expression in HH bees is greater than in LL bees.
